# Implementation Approaches to Strengthen a Food Insecurity Intervention for Older Adults in an Emergency Department

**DOI:** 10.1093/geroni/igaa057.123

**Published:** 2020-12-16

**Authors:** Aileen Aylward, Timothy Platts-Mills, Liane Wardlow, Conor Sullivan, Jessa Engelberg Anderson, Andrea Morris

**Affiliations:** 1 University of North Carolina at Chapel Hill, Chapel Hill, North Carolina, United States; 2 West Health Institute, La Jolla, California, United States; 3 ServiceNow, San Diego, California, United States; 4 West Health Institute, San Diego, California, United States

## Abstract

Food insecurity is prevalent among older adults, negatively impacts health, and may increase healthcare utilization. Emergency Departments (ED) are an important site of care for older adults. However, the feasibility of screening for food insecurity in EDs is unknown. We assessed the feasibility of implementing a screening and referral process to identify and address food insecurity among older adults in the ED and then monitored progress to overcome barriers to implementation. We developed a semi-structured interview (SSI) guide using the Consolidated Framework for Implementation Research. Prior to implementation, ED staff with diverse clinical backgrounds participated in SSIs. SSIs were analyzed using rapid analysis. Before and during implementation, we engaged hospital leadership to refine the screening and referral process. During implementation, we identified barriers through periodic reflections with staff, observing screenings, and reviewing Electronic Medical Record (EMR) data. Staff agreed that food insecure older adults would benefit from community services. Nursing Assistants (NA) were identified as key implementers. ED leaders expressed concerns about regulatory compliance, EMR integration, and NA scope of work, which were addressed. During implementation, barriers included competing priorities, lack of knowledge, and discomfort with the topic of food insecurity. Stakeholder input and reviewing EMR data led to adaptations including modifying criteria for referral and embedding training into NA orientation. Leadership and staff supported food insecurity interventions but identified several concerns. Steps to facilitate implementation included identifying staff to screen, EMR integration, and building staff efficacy. Reviewing screening data and soliciting stakeholder feedback enabled ongoing adaptations that strengthened implementation.

